# Novel In Vivo Mouse Cryoablation Model to Explore Unique Therapeutic Approaches for Premalignant Columnar Lesions

**DOI:** 10.3390/mps4010006

**Published:** 2021-01-05

**Authors:** Ana C. P. Correia, Danielle Straub, Silvia Calpe, Kausilia K. Krishnadath

**Affiliations:** 1 Center for Experimental and Molecular Medicine (CEMM), Amsterdam University Medical Center, 1105 AZ Amsterdam, The Netherlands; daniellestraub@hotmail.com (D.S.); s.calpe@amsterdamumc.nl (S.C.); 2 Department of Gastroenterology and Hepatology, Amsterdam University Medical Center, 1105 AZ Amsterdam, The Netherlands

**Keywords:** in vivo mouse model, cryoablation, columnar metaplasia, Barrett’s esophagus, stomach ablation, gastroesophageal disorders

## Abstract

Patients with epithelial metaplasias have an increased risk of developing malignancies. In Barrett’s esophagus, neo-columnar epithelium develops proximal to the squamous-columnar junction (SCJ) in the esophagus as the result of prolonged exposure to bile and acid reflux. Patients require lifetime periodic surveillance, due to lack of effective eradication therapies. The shortage of innovative treatment options is mostly attributable to the paucity of adequate in vivo models of neo-columnar epithelium regeneration. This protocol describes the generation of a cryoablation model to study regeneration of neo-epithelia at the SCJ. Cryoablation of the columnar and squamous mucosa at the SCJ was achieved through local application of liquid N_2_O in wild-type and reporter mice in combination with acid suppression. Acid suppression alone, showed restoration of the SCJ with normal histological features of both the neo-columnar and neo-squamous epithelium within 14 days. As a proof of principle, mice were treated with mNoggin, an inhibitor of bone morphogenetic proteins (BMPs), which are involved in the development of columnar epithelia. Local application of mNoggin to the ablated area at the SCJ significantly reduced the development of the neo-columnar mucosa. Although this model does not faithfully recapitulate the exact characteristics of Barrett’s esophagus, it is a well-suited tool to study the mechanisms of therapeutic inhibition of neo-columnar regeneration. It therefore represents an efficient and easy platform to test novel pharmacological therapies for treatment of neo-epithelial lesions at the SCJ.

## 1. Introduction

Metaplasia is the replacement of one tissue by a neo-tissue which is better adapted to its novel environment [1]. Metaplasia results from environmental stimuli, such as pH change, alcohol, smoking, and hormones, which cause chronic tissue inflammation and consequently the release of growth factors, cytokines, and trophic factors such as bone morphogenetic proteins (BMPs) [1,2,3]. Several of these factors promote tissue regeneration, progenitor cells may be activated and give rise to neo-epithelia, which are better adapted to the new environment [4]. Although metaplastic lesions are associated with an increased risk for malignant degeneration, at baseline, these lesions have almost normal physiological functions and benign histological features [1,5]. Due to persistent environmental conditions and ongoing inflammation, metaplastic lesions may become dysplastic and eventually progress to cancer [1,4,5].

Columnar metaplasia can be found in the distal esophagus at the squamo-columnar junction (SCJ) or in the glandular stomach [1]. Gastric intestinal metaplasia (GIM) and Barrett’s esophagus (BE) are examples of metaplastic lesions in the stomach and esophagus, respectively. GIM occurs mainly through atrophic gastritis caused by *H. pylori* infection and autoimmune gastritis [6]. These inflammatory processes induce a neo-epithelium (intestinal type of metaplasia) that resembles the small intestine [4,7]. In BE, the normal stratified squamous epithelium is replaced by columnar types of neo-epithelia, which can also acquire an intestinal phenotype in response to chronic bile and acid-related inflammation [4,8]. Inflammatory signals and trophic factors are released and responsible for the tissue repair [9]. BE refers to different types of neo-columnar epithelia, which are highly relevant since they can progress to dysplasia and terminate in highly malignant adenocarcinomas [4,10].

The current standard of care for metaplastic lesions relies on surveillance programs for the management of the metaplastic condition [11,12,13]. In case of dysplasia, endoscopic strategies are the treatment of choice, including ablative therapies and endoscopic resection [8,14,15]. Patients with metaplastic lesions in the stomach receive eradication therapy for *H. Pylori* in combination with acid suppression [16,17]. In case of non-dysplastic BE, patients are treated with acid suppression, by preference using proton pump inhibitors [18]. Acid suppressive therapies increase the pH in the stomach and decrease reflux, which reduces the inflammation of the mucosa [12,18]. Acid suppressive therapies do not lead to reversal or eradication of the metaplastic lesions. At present, there are no pharmacological therapies that can eradicate esophageal columnar metaplasia and used to improve the outcomes of ablative therapies [19]. Such therapies are of high importance given the fact that the incidence of columnar metaplasia and cancers associated with these lesions are rapidly increasing [20]. Ablative techniques are safe, well-tolerated, and with minimal complications, however around 10% of the patients have buried metaplastic cells leading to recurrence of the disease [21]. Thus, development of novel molecular targeted therapies which eradicate or prevent metaplastic lesions are highly desirable also to complement endoscopic strategies already implemented in the clinic. However, before the implementation of such therapies in the clinic, finding suitable preclinical models is of paramount importance.

BMPs and its natural antagonists, such as Noggin, are multifunctional signaling molecules, which are induced during tissue injury, inflammation, and tissue regeneration [22]. They also play important roles during embryonic development of, for instance, the gut and its mucosal lineages [9]. The role of these molecules in tissue regeneration in adult columnar mucosal tissues and in the development of metaplastic tissues has been studied more recently [23,24]. In mouse models BMP4 and its downstream pSMAD pathway, its antagonist Noggin, and targets have been demonstrated to be involved in the development of the earliest stages of columnar metaplasia [2,3,25]. Therefore, targeting the BMP pathway could be an attractive molecular strategy to intervene with the development of neo-columnar or metaplastic epithelium.

Herein, we describe a novel cryoablation mouse model to study and test the potential of therapies for prevention of neo-epithelia, which develops at the squamo-columnar junction in mice stomach. After we successfully established the novel mouse cryoablation model, we validated the model by targeting BMPs using a non-selective BMP antagonist, Noggin, that was locally applied.

## 2. Experimental Design

Cryoablation is performed at the SCJ in the mice stomach where the neo-epithelia develop. The mouse cryoablation model here described was established in CB6F1 wild-type mice and further validated in lineage tracing K5-GFP mice.

### 2.1. Materials

- Slip tip syringe with 26 G needle/vehicle solutions (BD, Franklin Lakes, NJ, USA; cat. no.: 309597);
- 5-0 vicryl absorbable suture/closure mouse muscle and skin (Ethicon, Somerville, NJ, USA; cat. no.: VCP303H);
- 5-0 Prolene no absorbable suture/for stomach opening exposure (Ethicon, Somerville, NJ, USA; cat. no.: 8720H);
- 8-0 Prolene no absorbable suture/closure mouse stomach (Ethicon, Somerville, NJ, USA; cat. no.: 8741H);
- Transpore^TM^ medical tape/mouse fixation (3M, St. Paul, MN, USA; cat. no.: 70200739582);
- Castroviejo needle holder (Fine Science Tools; 12060-01);
- Mayo scissor/cut skin (Fine Science Tools, Heidelberg, DEU; cat. no.: 14010-15);
- Fine scissors/cut stomach, muscle tissue (Fine Science Tools, Heidelberg, DEU; cat. no.: 14058-09);
- Adson forceps—DeBakey serrations/grasp tissues (Fine Science Tools, Heidelberg, DEU; cat. no.: 11616-15);
- Iris forceps/grasp tissues (Fine Science Tools, Heidelberg, DEU; cat. no.: 11064-07);
- Halsted-mosquito hemostats/needle holder for stomach opening exposure (Fine Science Tools, Heidelberg, DEU; cat. no.: 13009-12);
- Feeding needle/oral gavage of treatment (Fine Science Tools, Heidelberg, DEU; cat. no.: 18065-20);
- 100 × 100 mm gauze pads (B. Braun, Melsungen, DEU; cat. no.: BRAU9031324);
- 50 × 50 mm gauze pads (B. Braun, Melsungen, DEU; cat. no.: BRAU9031308);
- Cotton swaps (Heinz Herenz Hamburg, Hamburg, DEU; cat. no.: HERE1031318);
- Betadine^®^ solution/disinfect skin (Mylan, Canonsburg, PA, USA; cat. no.: NDC 67618-155-16);
- Sterile saline solution (NaCl 0.9%)/for injection and treatment dilutions (B. Braun, Melsungen, DEU; cat. no.: 1531131);
- Water for injection (B. Braun, Melsungen, DEU; cat. no.: 2351744);
- Baby milk powder (any commercial brand);
- Meloxicam/pain killer (Dopharma, Raamsdonksveer, NLD; cat. no.: 16A06-08C3);
- Baytril^®^ 25 mg/mL/antibiotic for the drinking water (Bayer, Leverkusen, DEU; cat. no.: 04007221042419);
- Omeprazole/anti-acid treatment (Teva Pharmaceuticals, Petah Tikva, ISR);
- Lubruthal/eye ointment (Dechra Pharmaceuticals, Northwich, GBR; cat. No.: 570117031-3291);
- SuperFrost Plus™ adhesion slides (Thermo Fisher Scientific, Waltham, MA, USA; cat. No.: J1800AMNZ).

### 2.2. Equipment

- Cage heating pad (Braintree Scientific, Braintree, MA, USA; cat. no.: HP 1M);
- Rodent anesthesia circuit;
- Induction chamber;
- Bead sterilizer (Harvard Apparatus, Holiliston, MA, USA; cat. no.: 72-0125);
- 1 mm Cryo-pen + liquid nitrous oxide (N2O) cartridge (Cryoalfa LUX, Radebeu, DEU; cat. no.: CA-L);
- Weight scale;
- Histology embedding station;
- Leica DM6 fluorescent microscope (Leica, Wetzlar, DEU);
- CryoStar NX50 cryostat (Thermo Fisher Scientific, Waltham, MA, USA).

### 2.3. Reagents

- Phosphate-buffered saline (PBS) (Thermo Fisher Scientific, Waltham, MA, USA; cat. no.: 10010023);
- Formalin solution, neutral buffered, 10% (Sigma-Aldrich, St. Louis, MO, USA; cat. no.: HT501128-4L);
- Ethanol (Sigma-Aldrich, St. Louis, MO, USA; cat. no.: 51976-500ML-F);
- Xylene (Sigma-Aldrich, St. Louis, MO, USA; cat. no.: 108298);
- Hematoxylin solution according to Mayer (Sigma-Aldrich, St. Louis, MO, USA; cat. no.: 51275);
- Eosin Y solution 1% (Sigma-Aldrich, St. Louis, MO, USA; cat. no.: 1170811000);
- DPX Mountant (Sigma-Aldrich, St. Louis, MO, USA; cat. no.: 06522-100ML);
- Tissue-Plus TM O.C.T. compound (Fisher Scientific, Waltham, MA, USA; cat. no.: 23-730-571);
- DAPI (4’,6-diamidino-2-phenylindole, dihydrochloride) (Thermo Fisher Scientific, Waltham, MA, USA; cat. no.: 62247);
- Recombinant mouse noggin protein (R&D Systems, McKinley, MN, USA; cat. no.: 1967-NG);
- Tamoxifen (Sigma-Aldrich, St. Louis, MO, USA; cat. no.: T5648).

### 2.4. Animals

All animal experiments were approved by the Animal Experimental Committee of the Amsterdam University Medical Center (Amsterdam UMC) and in compliance with the Animal Welfare Body (IvD), under the protocol number LEX159. All animals were kept at the Animal Research Institute of the Amsterdam UMC (ARIA), and procedures were performed under ARIA standard operating procedures (SOP).

All mice strains were housed in individual ventilated cages (+/+ IVC) in ventilated racks before, during, and after the cryoablation procedure. All mice used were older than 8 weeks with initial weights above 20 g.

The development of the cryoablation model was performed using wild-type CB6F1 mice purchased from Charles River (CB6F1/Crl strain code 176). Cryoablation protocol was validated by using K5-Cre-Rosa26-Tomato-GFP mice (K5-GFP).

C57BL/6J keratin 5 (K5) promoter green fluorescent protein (GFP) expressing mice (K5-GFP mice) were generated by crossing *ROSA26*-Cre reporter mice (*Gt(ROSA)26Sor^tm1Sor^*; JAX stock #007576), purchase from Jackson Laboratory, and *K5*-Cre mice (*Tg(BK5-CreERT*), kindly donated by Dr. Chen (Department of Life Sciences and Institute of Genome Sciences, National Yang-Ming University, Taipei, Taiwan) [26].

## 3. Procedure

### 3.1. Preoperative Care. Time for Completion: 3 Days (Not Accounting Mice Acclimatation)

**Figure 1:**
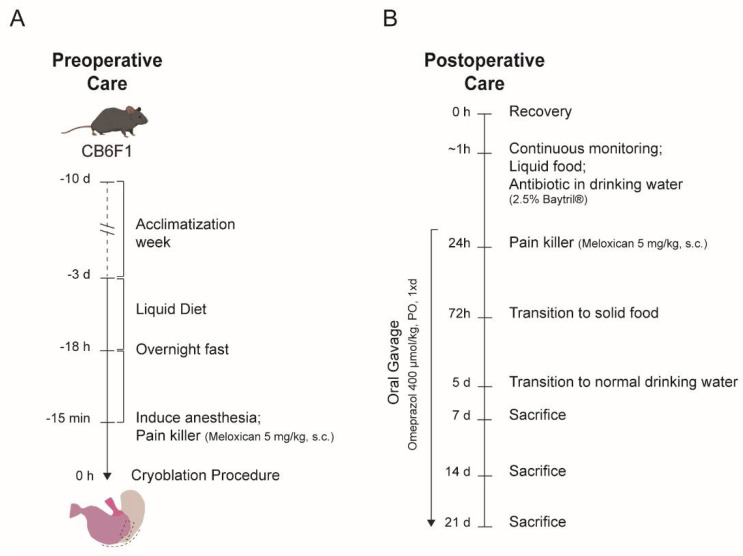
Schematic representation of the different procedural and animal care steps. (**A**) Timeline for preoperative cares and (**B**) postoperative animal care. s.c.: subcutaneously, PO: *Per os.*, oral administration.

1. Remove any solid pellet food from the +/+ IVC cages 3 days before the cryoablation procedure.
2. Replace the pellet food for liquid food (commercial baby milk prepared according to the supplier in drinking water).
3. Day prior to the cryoablation procedure (approximately 18 h prior), remove liquid food for overnight fasting (Figure 1A). Leave drinking water available ad libitum.

**NOTE**: If using K5-GFP mice, induce K5 positive cells by tamoxifen intra-peritoneal (i.p.) injection (10–12.5 mg/kg body weight) the day prior the ablation procedure.

### 3.2. Cryoablation Procedure. Time for Completion: 30 min per Mouse

**Figure 2:**
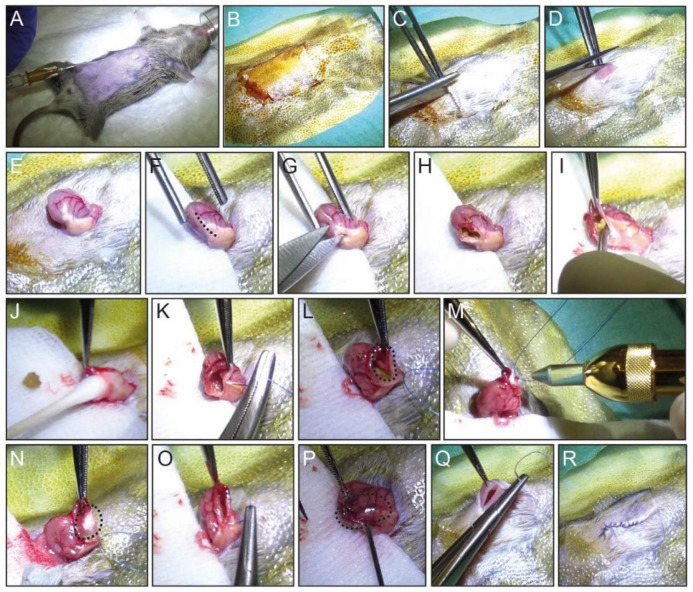
Step-by-step cryoablation procedure of the mouse stomach. (**A**,**B**) After shaving the abdomen, the mouse is gently fixed onto a heating pad with anesthesia through an inhalation cone. Pain killer is administered subcutaneously, and the skin disinfected. (**C**,**D**) A 2 cm midline vertical incision is made, and the underlying abdominal muscle is opened with scissors. (**E**) Reaching the stomach with the help of forceps, the stomach is outside the intraperitoneal cavity. (**F**,**G**) The stomach is opened by making and 0.75–1 cm incision in the stomach greater curvature, between the mouse forestomach and the glandular stomach. (**H**–**J**) The stomach content is removed with the help of forceps and a cotton swap. (**K**) One stitch is used to keep the stomach open for better visualization. (**L**,**M**) With proper visualization of the clean ablation area (dotted line), liquid N_2_O is applied onto the interior stomach wall at the SCJ. (**N**) Liquid N_2_O is applied 2× for 5 to 10 s each application. Frozen stomach wall (dotted line) is allowed to thaw before the second application. (**O**) The stomach is closed using an 8-0 non-absorbable suture. (**P**) The cryoablated area can be observed on the exterior of the stomach (dotted line). (**Q**,**R**) After placing the stomach back in the intraperitoneal cavity, the abdominal muscle and skin are closed.

1. In the non-sterile area, place the mouse in the induction chamber with 2% isoflurane. Under anesthesia, weigh the mouse and register his weight. Shave the abdominal area and transfer the mouse to the sterile field.
2. Mobilize the mouse in the sterile field with medical tape, place the anesthesia cone on the mouse nose, and apply eye ointment to prevent corneal damage (Figure 2A).
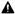
**CRITICAL STEP:** It is important to prepare the sterile field onto a heating pad (37 °C) to be able to maintain the mouse body temperature throughout the procedure.
3. Administer pain killer (Meloxicam, 5 mg/kg body weight) with a single subcutaneous (s.c.) injection with a 26 G needle syringe (Figure 2A).
4. Clean the skin with betadine solution (Figure 2B).
5. Start the surgical procedure by lifting the mouse skin with forceps and making a midline vertical incision on the abdomen, of about 2 cm (Figure 2C,D).
6. Use the round atraumatic forceps to reach the stomach inside the intraperitoneal cavity.
7. Pull the stomach out from the intraperitoneal cavity for better visibility and performance (Figure 2E).
8. Embed a sterile medical gauze pad with warm PBS and place it between the stomach and the mouse skin (Figure 2F).
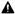
**CRITICAL STEP:** The use of sterile gauze pad is important to avoid direct contact between the two distinguish surfaces, stomach and skin. In addition, it is essential when removing the stomach content in step 10. This step will minimize possible infection post-surgery.
9. Using a fine scissor, make an incision of 0.75 to 1 cm long in the middle of the greater curvature of the mouse stomach including the SCJ as shown in Figure 2F,G.
10. Once opened, remove the stomach content with the help of round-tip forceps and cotton swaps until the area at the SCJ for the ablation is clean (Figure 2H–J).
11. For better visualization of the stomach interior, make a stich with a 5/0 non-absorbable suture and hold it with a needle holder and set it aside (Figure 2K).
12. With a proper visualization of the ablation area (Figure 2L, dotted line), use the cryo-pen to apply liquid N_2_O onto the anterior wall of the mouse stomach right in the SCJ (Figure 2M,N).
13. Apply liquid N_2_O twice in the same area for 5 to 10 s each time, waiting for the tissue to thaw in between applications.
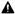
**CRITICAL STEP:** The time of each liquid N_2_O application depends on how stretched the mouse stomach is, i.e., how thin the stomach wall is. It is important not to exceed the duration of 10 s and pause briefly between applications to assess the damage and avoid perforation of the stomach wall.
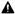
**WARNING:** Cryoalpha pen allows for precise and accurate applications of liquid N_2_O, which can produce temperatures of −89 °C. Cryoalpha pen is a safe instrument and should be held like a pencil when in use, only aiming the applicator to the area of interest.
14. Close the stomach using an 8/0 non-absorbable suture with continuous stiches (Figure 2O). The ablated area can be observed on the exterior of stomach by a darker red color of the wall (Figure 2P, dotted line).
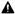
**CRITICAL STEP:** Carefully inspect if the stomach is completely closed. Small openings in the mouse stomach can lead to leakage of the stomach content into the peritoneal cavity.
15. Place the ablated stomach back inside the intraperitoneal cavity and close the abdominal wall and skin separately by using and a 5/0 vicryl absorbable suture (Figure 2Q,R).

**NOTE**: Troubleshooting of different problems, reasoning, and solutions, that can be encountered during and after the cryoablation procedure can be found in Table 1.

### 3.3. Postoperative Care. Time for Completion: Up to 21 Days

1. Remove the mouse from the sterile field and place the mouse back to the +/+IVC cage in the ventilated rack onto a warm pad for the first 24 h postoperative hours.
2. Add liquid food (baby milk) in the +/+ IVC cage and antibiotic (Baytril^®^, 25 mg/mL) in the drinking water
3. Observe the mouse recovery from the anesthesia and register any signs of discomfort.

**NOTE:** On day 1 after the cryoablation procedure, administer a single s.c. injection of pain killer (5 mg/kg body weight) in case of visible signs of discomfort and a single s.c. injection of saline solution (200–300 µL) when signs of dehydration are observed.

4. **NOTE:** Animals need to be euthanized immediately if a humane endpoint is reached after surgery or during the follow up period. In our institution, humane endpoints include severe weight loss (>15% body weight loss compared to the initial weight, an extended period of weight loss without improvement after 48 h) or severe dehydration for more than 72 h without improvement after s.c. saline injections.
5. From day 1 post-cryoablation and throughout the experiment, give daily oral gavage of anti-acid (Omeprazole, 400 µmol/kg).
6. On day 3 post-cryoablation, replace liquid food by normal pellet food ad libitum.
7. On day 5 post-cryoablation replace antibiotic water by tap water ad libitum.

### 3.4. End of the Experiment. Time for Completion: Approximatly 30 min per Mouse

**Figure 3:**
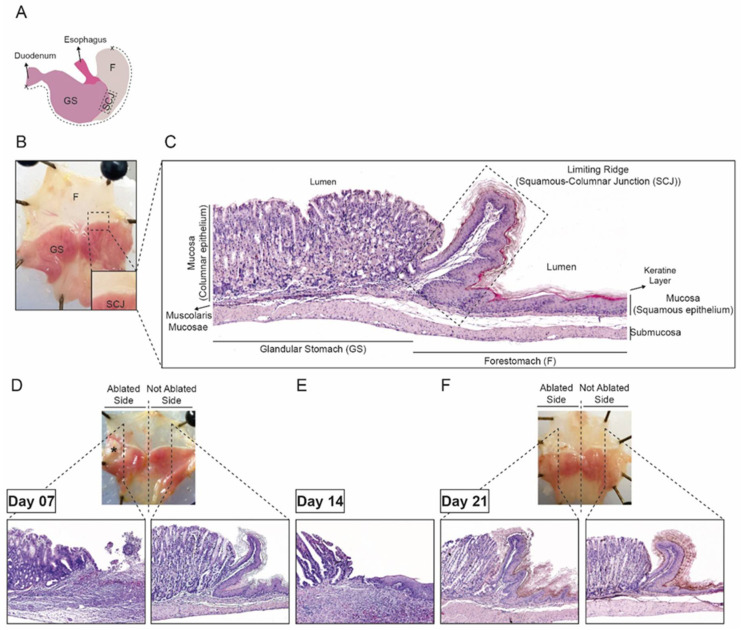
Results of cryoablation of the squamous-columnar junction (SCJ) in mice stomach. (**A**) Schematic representation of a mouse stomach with an indication of the different stomach compartments. The dotted line indicates the greater curvature along which the stomach was opened for performing the ablation and analysis. (**B**) Anatomy of not ablated control CB6F1 mouse stomach that has been opened along the greater curvature, with the demonstration of the location of the SCJ. (**C**) Hematoxylin and eosin (HE) staining of a normal control mouse with description of the different histological parts of the mouse stomach. (**D**) Anatomy of CB6F1 mouse 7 days after cryoablation, with HE staining of the mouse stomach of the cryoablated side (left) and not ablated area (right). (**E**) HE staining of CB6F1 mouse stomach from the ablated side, 14 days after cryoablation. (**F**) Gross morphology of CB6F1 mouse 21 days after cryoablation, with HE staining of the mouse stomach in the cryoablated side (left) and not ablated area (right). F, forestomach; GS, glandular stomach; SCJ, squamous-columnar junction.

1. Cull the mice at the pre-determined experimental endpoints (t = 7, 14, and 21 days) by placing the mice in a CO_2_ chamber.
2. Immediately after culling, remove mice from the CO_2_ chamber, shave the abdominal area, and open using a round tips scissor by making an upper-midline vertical incision of the abdomen.
3. Dissect and isolate the stomach.
4. Holding the isolated stomach, with the stomach curvature upwards, open the stomach from the duodenum opening to the forestomach part along the greater curvature (Figure 3A).
5. Remove the stomach content and clean with PBS.
6. Pin the open stomach to a paraffin block to flatten the stomach for histological preparation.

### 3.5. Histological Preparation

#### 3.5.1. Tissue Fixation and Slide Preparation. Time for Completion: 3 Days

1. Fix the pinned stomach into 10% neutral buffered formalin solution for 24 h.
2. Transfer the tissues to labeled histology cassettes and dehydrate the tissues in a series of increasing ethanol percentage solution (70%, 80%, 90%, and 96% ethanol) until 100% ethanol, during 1 h for each ethanol grade.
3. Clean tissue cassettes in 100% xylene for 1 h.
4. Place the cleaned tissue cassettes in a high-temperature paraffin bath (60 °C) overnight.
5. Transfer the cassettes to an embedding station and cut the fixed tissue in four pieces as represented in Figure 3D,F.
6. Place tissue pieces in a mold and embed it in paraffin.
7. Cut the tissue paraffin blocks as 5 µm sections and place onto a slide.
8. Dry the tissue slides at 37 °C for at least 16 h.

#### 3.5.2. Hematoxylin and Eosin Staining. Time of Completion: 2 h and 30 min

1. Deparaffinize the tissue slides in 100% xylene for 10 min.
2. Rehydrate the slides in a graded series of decreasing ethanol percentage solutions (100%, 96%, 90%, 80%, and 70%) for 10 min each grade.
3. Place the slides in hematoxylin staining solution for 10 min at room temperature.
4. Wash slides in running water for 5 min.
5. Counter stain with 1% eosin solution for 5 min.
6. Dehydrate the tissue slides with increase grade of ethanol percentage (70%, 80%, 90%, 96%, and 100%) for 10 min each grade.
7. Clean the slides with 2× xylene for 2 min each.
8. Let the slides dry in the flow cabinet and mount with DPX mounting medium and a coverslip.

#### 3.5.3. Tissue Cryopreservation. Time of Completion: 10 min per Mouse Stomach

1. Cut the pinned stomach from K5-GFP mice into 4 pieces (<5 mm) as represented in Figure 3D,F.
2. Place the tissue pieces flat inside a tinned-metal sample container or a cryovial.
3. Snap frozen in liquid nitrogen and store the samples at −80 °C until further analysis.

#### 3.5.4. Cryo Slide Preparation and Fluorescence Microscopy: Time of Completion: 30 min per Sample

1. In a CryoStat station, remove the cryopreserved tissue and place it onto a cold base.
2. Cover the tissue with cryo-embedding media (OTC).
3. Place the mold in the Peltier fast-freezing platform of the cryostat to ensure the tissue is frozen before sectioning.
4. Cut the frozen tissue block into 6 µm sections and place onto a Superfrost Plus adhesion glass slide.
5. Dry the slides at room temperature and store them in a slide box at −20 °C for later use.
6. Stain the slides with DAPI (1:1000) for nuclear staining and visualize under a fluorescent microscope.

## 4. Expected Results

### 4.1. Establishment of the Cryoablation Mouse Model

To establish the cryoablation model, the SCJ of the stomach in wild-type CB6F1 mice was ablated and histologically analyzed at day 7, 14, and 21 post-cryoablation on hematoxylin and eosin (HE) staining tissue slides. The mouse stomach consists of two main compartments, the forestomach, lined with multilayered keratinizing squamous epithelium and the glandular stomach, lined with single layered columnar epithelium. The two compartments are delineated by the SCJ as represented in Figure 3B,C. All mice received proton pump inhibition after the ablation and daily during the healing process. When compared to the not ablated side of the stomach, we observed that 7 days after the ablation the SCJ was damaged and inflamed, and both the columnar and squamous cells were absent, indicating the success of the cryoablation to eliminate both type of epithelia at the SCJ (Figure 3D). Fourteen days post-cryoablation, we found regeneration of normally appearing neo-squamous and neo-columnar proximal and distal to the SCJ in approximately 60% of the cryoablated mice. Representative HE stainings of nearly completed regenerated cryoablated mouse stomach at day 14 is demonstrated in Figure 3E. After 21 days, the ablated epithelia were completely regenerated with neo-epithelia and their histology was comparable to that of the not ablated stomach side (Figure 3F). Macroscopic analysis of surrounded tissues after culling, didn’t show any tissue injury was observed at 14 and 21 days post-cryoablation.

### 4.2. Intervening with the Regenerative Process of the Neo-Columnar Epithelium at the SCJ

**Figure 4:**
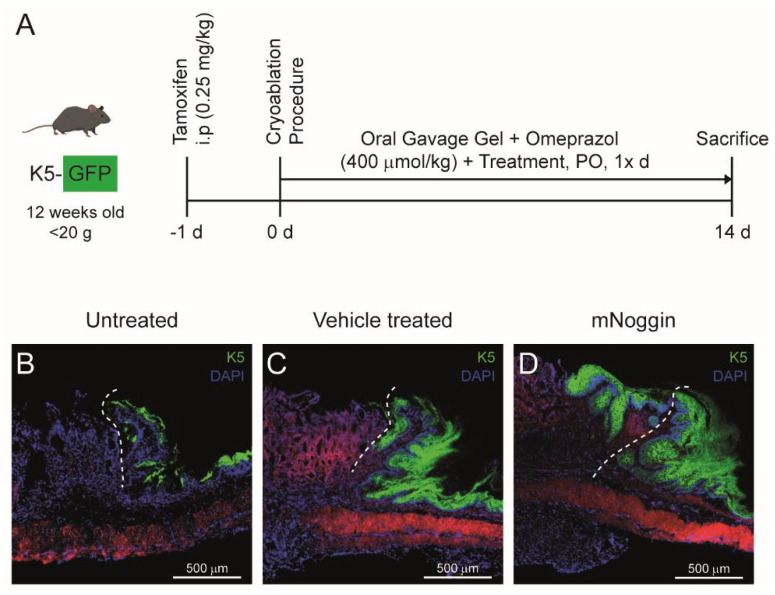
Preliminary results using the cryoablation procedure. (**A**) The experimental set-up of the validation of the cryoablation procedure is according to Figure 1 with minor adjustments. (**B**) Fluorescent image of untreated K5-GFP mice stomach. (**C**) Fluorescent image of vehicle-treated K5-GFP mice, 14 days post-cryoablation. (**D**) Fluorescent image of K5-GFP mice, 14 days post-cryoablation, treated with mNoggin (orally, 1× day). The delimitation of the original SCJ that separated the squamous and the columnar epithelium before the ablation is indicated by the dotted white line. i.p.: intraperitoneal injection; PO: *Per os.*, oral administration.

We performed a proof-of-principle study to validate the cryoablation model by testing an unselective inhibitor for bone morphogenetic proteins (BMPs), Noggin, which inhibits BMP2 and BMP4, among other BMPs [27]. BMP proteins are critical for columnar cell regeneration and the homeostasis of columnar epithelia [28]. For this purpose, K5-GFP lineage-tracing mice were used to trace the proliferation of K5 positive cells while mice were treated with either vehicle (control) or with vehicle plus mouse Noggin (mNoggin) (Figure 4A). We observed that in cryoablated vehicle-treated K5-GFP mice, the renewal of epithelium in the SCJ is similar to the not cryoablated untreated K5-GFP mice. The number of K5 positive cells was higher and cells more intensely expressed K5-GFP in the cryoablated vehicle-treated K5-GFP mice. This is probably due to the rapid proliferation of K5 positive squamous (stem) cells of the cryoablated region of the stomach to regenerate the squamous mucosa (Figure 4B,C). In the mNoggin treated K5-GFP mice, regeneration of the columnar region failed and instead a neo-squamous tissue was observed similar to the squamous region in control mice. In addition, the squamous region extended to the ablated columnar zone distally of the SCJ. The regeneration of columnar epithelium was therefore inhibited and was replaced by squamous epithelium. Thus, it seems that Noggin inhibits the regeneration of the columnar epithelium by inhibiting several BMPs, while the regeneration of the squamous epithelium is not affected (Figure 4D).

## 5. Conclusions

To date, due to the lack of good pre-clinical models, no promising molecular targeting therapies were developed for premalignant columnar lesions. Here, we developed a novel mouse model using wild-type and genetically modified mice. This model allows to study the cellular changes and regeneration process that occur upon cryoablation of epithelial tissue at the SCJ. We developed this model as a platform to be able to study potential therapies for the treatment of neo-epithelia that occurs at the SCJ in stomach and esophagus. We believe that the model can also be translated to other organs in which neo-epithelia develop in transitional zones of squamous to columnar mucosa, but further validation will be necessary. Ablative therapies are frequently used for treatment of epithelial lesions, for instance, as applied in the esophagus for treatment of Barrett’s esophagus. In our set up, we complemented the ablative procedure with an inhibitor of BMPs to illustrate the effects on the regeneration of columnar epithelium at the SCJ. In our protocol, this inhibitor (mNoggin) was locally applied to prevent systemic off target effects. The cryoablation surgical model described here is a viable model, where data material can be obtained in a reasonable timeline of about 2 to 3 weeks, after starting the experiment. This procedure was successful in two different mouse models, and we believe that it can also be used in other mouse models and applied to other organs. Both CB6F1 and lineage tracing K5-GFP mice showed similar recovery time and recuperating to their normal histology after cryoablation.

In our protocol, mice were treated with acid suppression medication (PPIs—proton pump inhibitors) to reduce the acidic environment in the stomach of the mice. We believe that this approach, together with a targeted molecular therapy, will be required in the clinic for a better and efficient regeneration of the neo-epithelium, as shown in our animal model.

Targeting BMP signaling revealed to be an effective molecular candidate for the treatment of columnar metaplasia at the SCJ. To our knowledge, there are no other targeted therapies specifically developed for columnar pre-neoplastic lesions. Noggin is a non-selective BMP inhibitor, and given its pleiotropic effects, it is not suitable for systemic treatment in humans. Recently, we developed novel highly selective inhibitors against BMP2 and BMP4 [29,30]. Future studies will demonstrate the efficacy and safety of these novel anti-BMP therapies and their use for treatment of BE.

In sum, this novel in vivo model shows to be a potential tool to test promising targeting drugs, for instance, to complement the ablative therapies used for the treatment of metaplasia and neo-epithelia, including Barrett’s esophagus.

## Figures and Tables

**Table 1:** Troubleshooting for different problematic scenarios during and after cryoablation.

Problem	Procedure Step	Possible Reason	Solution
Stomach perforation	Figure 2M	Extensive exposure to liquid N_2_O	This step needs to be adjusted depending on the animal model used. K5-GFP mice are more fragile than CB6F1, therefore the time of exposure needs to be slightly less than with the CB6F1 mice (2× 5 to 8 s for K5-GFP and 2 × 8 to 10 s for CB6F1 mice). Make sure that the stomach is outside the abdominal cavity to avoid exposure of other vital organs to liquid N_2_O.
Bleeding from the stomach	Figure 2O	Cryoablation of the stomach	It is normal to observe some minimal bleeding after cryoablation.
Bleeding from the stomach	Figure 2G	Damage of the stomach vasculature	Make sure to open the stomach at the greater curvature, where there is less vasculature. Apply pressure using a cotton swab for approximately 1 min on the bleeding spot or blood vessel.
Bleeding from the stomach	Figure 2I–O	Forced grabbing of the stomach wall during ablation	Use appropriate forceps to hold the stomach wall during cryoablation. Holding too tight will damage the stomach wall.
Leakage from the stomach incision	Figure 2P	The stomach is not sutured properly	Additional stitches are required.
Not drinking liquid food	Postoperative care (day 0–2)	It is not unusual that some animals dislike liquid food	Provide small amounts of solid food after day 2 or soften the pellet food with water.
Not eating solid food and low mobility	Postoperative care	High discomfort	Extreme weight loss can be an indication of infection due to perforation of the stomach and exposure of stomach content to the abdominal cavity. Endpoint rule applies and the animal should be euthanized.
Dehydration	Postoperative care	Not drinking water due to discomfort	s.c. injection of saline solution.
Opening of the skin wound	Postoperative care	Animals can reach and eat the stitches	Additional stitches or other means are required to close the skin incision/wound.
